# Exploring cutting-edge approaches in diabetes care: from nanotechnology to personalized therapeutics

**DOI:** 10.1007/s00210-024-03532-7

**Published:** 2024-10-25

**Authors:** Gihan F. Asaad, Ahmed S. Doghish, Ahmed A. Rashad, Walaa A. El-Dakroury

**Affiliations:** 1 Department of Pharmacology, Medical Research and Clinical Studies Institute, National Research Centre, Giza, Egypt; 2 Department of Biochemistry, Faculty of Pharmacy, Badr University in Cairo (BUC), Badr City, Cairo, 11829 Egypt; 3 Department of Biochemistry and Molecular Biology, Faculty of Pharmacy (Boys), Al-Azhar University, Nasr City, Cairo, 11651 Egypt; 4 Department of Clinical Pharmacy and Pharmacy Practice, Faculty of Pharmacy, Badr University in Cairo (BUC), Badr City, Cairo, 11829 Egypt; 5 Department of Pharmaceutics and Industrial Pharmacy, Faculty of Pharmacy, Badr University in Cairo (BUC), Badr City, Cairo, 11829 Egypt

**Keywords:** Diabetes mellitus, Drug delivery, Nanoparticle, Insulin, Gene therapy

## Abstract

Diabetes mellitus (DM) is a persistent condition characterized by high levels of glucose in the blood due to irregularities in the secretion of insulin, its action, or both. The disease was believed to be incurable until insulin was extracted, refined, and produced for sale. In DM, insulin delivery devices and insulin analogs have improved glycemic management even further. Sulfonylureas, biguanides, alpha-glucosidase inhibitors, and thiazolidinediones are examples of newer-generation medications having high efficacy in decreasing hyperglycemia as a result of scientific and technological advancements. Incretin mimetics, dual glucose-dependent insulinotropic polypeptide, GLP-1 agonists, PPARs, dipeptidyl peptidase-4 inhibitors, anti-CD3 mAbs, glucokinase activators, and glimins as targets have all performed well in recent clinical studies. Considerable focus was placed on free FA receptor 1 agonist, protein tyrosine phosphatase-1B inhibitors, and Sparc-related modular calcium-binding protein 1 which are still being studied. Theranostics, stem cell therapy, gene therapy, siRNA, and nanotechnology are some of the new therapeutic techniques. Traditional Chinese medicinal plants will also be discussed. This study seeks to present a comprehensive analysis of the latest research advancements, the emerging trends in medication therapy, and the utilization of delivery systems in treating DM. The objective is to provide valuable insights into the application of different pharmaceuticals in the field of diabetes mellitus treatment. Also, the therapeutic approach for diabetic patients infected with COVID-19 will be highlighted. Recent clinical and experimental studies evidence the Egyptian experience. Finally, as per the knowledge of the state of the art, our conclusion and future perspective will be declared.

## Introduction

Diabetes mellitus (DM) affects 400 million individuals globally(Khursheed et al. 2019) and has increased in prevalence by 50% in the last decade (Cheng et al. 2021). Globally, 9.3% (463 million) of people have diabetes, which is anticipated to climb to 10.9% (700 million) by 2045 (Saeedi et al. 2019). In some respects, this modern epidemic is surprising, considering that diabetes is one of the oldest diseases on the globe, having been documented in historical documents from civilizations as far back as ancient Egypt, India, and Persia (Mohajan 2023; Singh et al. 2023). DM is a metabolic condition defined by hyperglycemia caused by insulin deficiency, pancreatic cell damage, or insulin resistance due to a lack of insulin utilization (Ciochina et al. 2022). This metabolic disorder has potentially lethal long-term macrovascular, microvascular, and neuropathic effects. The most prevalent kinds of diabetes are type 1 and 2; however, diabetes can also occur during pregnancy and in conjunction with other illnesses such as drug or chemical toxicity, genetic abnormalities, endocrinopathies, insulin receptor (IR) problems, and pancreatic exocrine disease (Antar et al. 2023).

DM is divided into two categories, each with its own set of causes. In type 1 DM (T1DM), the immune system damages the pancreas cells it is supposed to protect because of a genetic predisposition for doing so. Elevated hepatic glucose synthesis, poor insulin secretion, or abnormalities at the receptor and post-receptor levels may all contribute to type 2 diabetes (TIIDM), which occurs when the body either does not respond normally to insulin (insulin insensitivity) or does not produce sufficient insulin (insulin resistance) (Lin et al. 2021). Type III DM encompasses a wide variety of other forms of diabetes with well-established causes. These include diabetes caused by monogenic conditions, genetic abnormalities, a dysfunctional pancreas, endocrinopathies, infections, hereditary syndromes, and even certain drugs. Pregnancy-related diabetes is considered type IV diabetes (GDM) (McIntyre et al. 2019). There are many factors linked to an increased risk of diabetes (Ismail et al. 2021), such as neonatal and young-onset diabetes, which are the two most common types of monogenic diabetes that result from mutations in a single gene (Bonnefond et al. 2023). Johns et al. (2018) declared that gestational diabetes develops as a result of hormonal changes that occur throughout pregnancy. The placenta secretes substances that make cells more resistant to insulin’s effects. Moreover, Ode et al. (2019) added that cystic fibrosis causes thick mucus to build up in the pancreas, preventing it from producing enough insulin. One of the main contributors to diabetes is hemochromatosis, a condition in which the body stores too much iron, and can build up in the pancreas (Barton and Acton 2017). In addition to this, some hormonal disorders cause the body to create excessive hormones, i.e. cortisol and growth hormone, which can lead to insulin resistance and diabetes (Barbot et al. 2018). Not only hormonal disturbance can lead to diabetes, however. Pancreatic cancer, pancreatitis, or trauma can destroy the beta cells or reduce their ability to produce insulin (Wei et al. 2020; Zhao and Liu 2020; Wayne et al. 2024). What also causes the appearance of the disease is drugs that are either abused or used correctly, yet the drug has a bad risk over the pancreas. Certain drugs have the potential to harm beta cells or cause them to malfunction. Niacin, some diuretics, anti-seizure pharmaceuticals, psychiatric meds, HIV treatments, pentamide, glucocorticoids, anti-rejection therapies, and statins are among these medications (Kaur and Mharazanye 2024).

## Pathophysiology of DM

In the intestine, SGLT1 and glucose transporter-2 (GLUT-2) absorb complex dietary carbs. After the entrance of glucose via glucose transporters, it is phosphorylated via glucokinase and then metabolized to pyruvate during glycolysis. The pyruvate mitochondrial oxidation results in intracellular ATP accumulation, which activates the plasma membrane depolarization due to the closure of ATP-dependent potassium (K^+^) channels, reducing K^+^ efflux and leading to full depolarization of the voltage-gated Ca^2+^ channel, resulting in the release of insulin (Walczewska-Szewc and Nowak 2021). Cells may be harmed by autoimmunity or for unknown causes, resulting in a lack of insulin production in young people with maturity-onset diabetes, while neonatal diabetes may result in a lack of glucose sensing (Yau et al. 2021). At the cellular level, insulin interacts with a subunit of the IR. Phosphoinositide 3-kinase (PI3K) is turned on after phosphorylation of the IR substrate. The phosphatidylinositol 4,5-bisphosphate is then converted resulting in the creation of active protein kinase B.

Akt phosphorylates the cap of GLUT-4, which is carried onto kinesin motors and anchored on the cell surface, enabling glucose to enter. When there is a shortage of insulin, as in TIDM, this pathway cannot be activated. When any of the following phosphorylation processes is disrupted, insulin resistance develops, leading to TIIDM (Lee et al. 2022). Chyme releases GLP-1 and GIP when food reaches the colon. Even before the food is digested, this stimulates the pancreas to boost insulin release and decrease glucagon production. The dipeptidyl peptidase 4 (DPP-4) enzyme metabolizes physiological GLP-1.

## Therapeutic targets for DM (ominous octet)

In DM, the medication targets are often referred to as ominous octets (Defronzo 2009; Chawla et al. 2020). At first, there is an insulin secretion deficiency from Langerhans β cells. Later, Langerhans α cells release more glucagon, and this leads to increased hepatic glucose production. These effects might cause an impairment of neurotransmitters in the brain, which then might increase lipolysis, increase kidney reabsorption of glucose, and reduce the small intestine incretin effect. Finally, these steps could lead to insufficient liver, skeletal muscle, and fat glucose uptake.

The isolation of insulin from the islets of a canine pancreas by Banting and Best in 1921 (De Leiva-Hidalgo et al. 2020) sparked the development of anti-diabetic medications. Insulin became commercially available in the United States soon after (Gerstein and Rutty 2021). Scientists have advanced recombinant DNA technology to generate analogs for insulin by changing the insulin side chains in the modern day (Donner 2000). Several novel pharmaceuticals, such as sulfonylureas and sodium-glucose co-transporter-2 inhibitors (SGLT2i), entered the market after 1956 (Su et al. 2023). Cardiovascular outcome trials (CVOTs) became essential after the rosiglitazone alert to establish the antidiabetics’ safety. Since then, major adverse cardiovascular events (MACEs) have become significant effectiveness endpoints for antidiabetics, in addition to hemoglobin A1c (HbA1c) (Cefalu et al. 2018).

## Treatment approach for DM

### Insulin-based treatment for TIDM and TIIDM

#### Insulin and insulin analogs

**Fig. 1:**
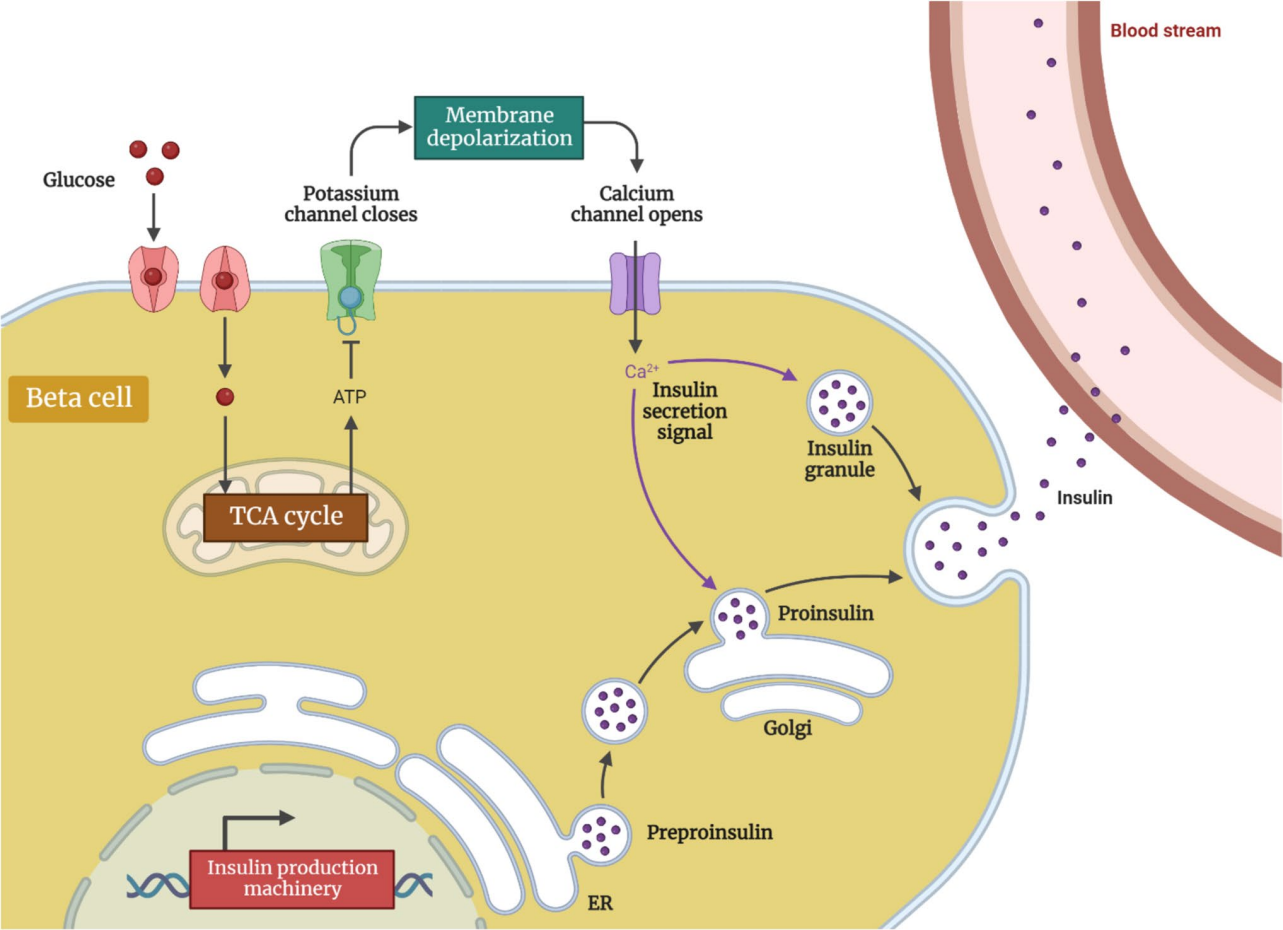
Insulin production pathway

Insulin is made up of two parts: preproinsulin and proinsulin. After that, proinsulin is transformed into insulin (Fig. 1), and C-peptide is stored in secretory granules, ready to be released as needed. Insulin are used to treat all types of TIDM, as well as particular TIIDM cases (Seetharaman et al. 2022). There are rapid-acting insulin (regular insulin and semilente), sustained-release insulin (lente and isophane), and ultra-long-acting insulin (ultralente).

The bolus and basal versions are used for administration(Donner 2000). Intensive insulin therapy has been shown in multiple studies to extend C-peptide generation in newly diagnosed TIDM patients (Seetharaman et al. 2022). Recent evidence from real-world outcomes research, however, suggests that insulin alone is unsuccessful for TIIDM patients and should be supplemented with additional antidiabetic medications (Feng et al. 2024).

#### Types of insulin preparations

Insulin analogs include both ultra-short like aspart, lispro, and glulisine (Table 1) and ultra-long like detemir, glargine, and degludec (Cernea and Raz 2020). Admelog and basiglar, respectively, are biosimilars of glargine and lispro that were approved (White and Goldman 2019). Insulin icodec is a novel insulin analog given once weekly. It was assessed in recent phase-3 research. It possesses a strong binding to albumin (HÖVELMANN et al. 2020; Nishimura et al. 2020). Icodec reduced HbA1c to the same extent as U100 glargine. Even though there were more incidences of clinically severe hypoglycemia in the icodec group, this was not statistically different from the glargine group (Nishimura et al. 2020).

**Table 1:** Ultra-short and short-acting insulin

	Ultra-short acting insulin (lispro, aspart, glulisine)	Short-acting (regular) insulin (humulin R, novolin R)
Uses	Similar to regular insulin (designed to be better than regular insulin)	Designed to (1) Control postprandial hyperglycemia (2) Treat emergency diabetic ketoacidosis
Chemical structure	Monomeric analogue	Hexameric analogue
Time of administration	S.C 5–15 min before meal	S.C 30–45 min before meal Forms insulin depot Hexamers break into dimers and finally into monomers
Onset of action	0–15 min (S.C)	30–45 min (S.C)
Peak serum levels	30–90 min	120–240 min
Duration of action	3–4 h	6–8 h
Usual administrations	2–4 times/day or more	2–3 times/day or more

#### Insulin therapy complications

The complications of insulin therapy include severe hypoglycemia that is less than 50 mg/dl (that might be due to a meal being missed, excessive (unusual) physical exercise, or overdose of insulin), weight gain, insulin resistance, or local or systemic allergic reactions.

Modi et al. suggest that the presence of excipients like phenol and meta-cresol in human insulin may be to blame for the latter reactions. If highly concentrated insulin were used, the amount of these excipients may be reduced, hence reducing the chance of allergic reactions(Cernea and Raz 2020).

#### Recent insulin and insulin delivery pumps

As an alternative to conventional insulin injections or insulin analogs, premix insulin are often employed in a basal-bolus or split-mix insulin regimen (Dawoud et al. 2023). Since degludec-aspart consists of the deludec (longest-acting) and the aspart (shortest-acting), it is considered as the optimal combination(Rendell 2019). Intranasal insulin, jets, pens, oral, rectal, ocular, buccal insulin, and transdermal insulin are some of the more recent insulin delivery systems (Bahman et al. 2019). W.A. El-Dakroury et al. established an intranasal carbopol gel to administer insulin. Furthermore, it increased the release of insulin and intranasal absorption in rabbits while also delivering a prolonged controlled hypoglycemic effect (El-Dakrouri et al. 2010). Regarding insulin delivery pumps, this technique ensures that TIDM and TIIDM patients receive extensive insulin therapy. Hybrid closed-loop insulin systems, sometimes known as “artificial pancreas,” are completely automated devices that provide insulin on their own ((McAdams and Rizvi 2016; Sora et al. 2019). The US FDA recently approved two of these devices from Medtronic and Tandem. The development of a third device, Do-It-Yourself, is presently underway. The security of these devices has improved in recent years, with a lower danger of being hacked (Seetharaman et al. 2022).

### Recent management for T1DM

#### Immunotherapy for T1DM (anti-CD3 monoclonal antibody)

Treatment with anti-CD3 mAb is considered the most immunotherapy for TIDM. In a randomized phase-1/2 study, individuals with a recent diagnosis of TIDM were given teplizumab for 14 days. C-peptide responses and insulin production were shown to be maintained for a total of 2 years.

A mixed-meal tolerance assessment, serum C-peptide, and glycemic control were the primary objectives of a phase-2b study with otelixizumab in individuals with TIDM. Otelixizumab 9 mg showed the best metabolic response while doses over 9 mg increase the risk of cytokine release in addition to a lower response than that obtained by 9 mg (Mignogna et al. 2021). The positive effects occurred only in patients functioning β cell residuals.

#### Theranostics MRI

It means that two substances, diagnostic imaging agents and medicines, are combined and delivered to the patient as a single agent. It aids in the determination of site localization, examination of bio-distribution, determination of the ideal dosage, and monitoring of patient responses after receiving this medication. A protected graft copolymer (PGC) containing gadolinium-loaded nanoparticles is used for diagnosis. In vivo, MRI revealed that this PGC is accumulated at the site of leaky blood arteries in the islets of diabetic mice. It emphasizes the changes in diabetes islet vascular permeability and blood volume. Gadolinium can be replaced by superparamagnetic iron oxide nanoparticles, which can also aid in the detection of vascular leakage in conjunction with inflammation. Fatty acid–containing PGC aids in the delivery of GLP-1 to β-cells, inhibiting apoptosis and stimulating the production of β-cells. PGC also aids in the stabilization and prolongation of endogenous GLP-1 half-life, allowing it to reach the pancreatic islets (Roy et al. 2021).

#### Non-insulin-based treatment for TIIDM

Insulin monotherapy is shown to be ineffective for TIIDM patients, necessitating the use of other antidiabetics to achieve optimal results. There are now several formulations of these drugs on the market. In terms of their effects on insulin, drugs may be classified as either insulin secretagogues, insulin sensitizers, or glucose regulators (Zhao et al. 2020).

### Insulin sensitizers

#### Biguanides (metformin)

When oral monotherapy fails to control type 2 diabetes, biguanides are the initial therapeutic option. Biguanides may be administered alone or in conjunction with insulin secretagogues or thiazolidinediones.

It is the first-line treatment for TIIDM, and it can be used alone or in combination with insulin secretagogues or thiazolidinediones in type 2 diabetics for whom oral monotherapy is inadequate.

The mechanism of action of biguanides is that it increases the sensitivity of insulin, antagonizes glucagon-mediated cyclic adenosine monophosphate (cAMP) production (Wang et al. 2021), inhibits hepatic gluconeogenesis, slows intestinal absorption of sugars, and improves peripheral glucose uptake, and decreases appetite which leads to a lowering of the patient’s weight.

The advantages of biguanides over insulin and secretagogues might include that metformin does not promote weight gain or provoke hypoglycemia, reduces plasma triglycerides by 15 to 20%, and, apart from insulin, identifies as the treatment option for GDM by the recommendation of the Royal College of Obstetricians and Gynaecologists (Kaur et al. 2019).

The main adverse effects of biguanides include anorexia, nausea, vomiting, diarrhea, abdominal discomfort, and lactic acidosis (Kwon et al. 2020). As a result, it should be avoided by people with renal disorder, hypotension, lung disease, heart failure, or hepatic disease (Haq et al. 2021).

#### Thiazolidinediones

**Fig. 2:**
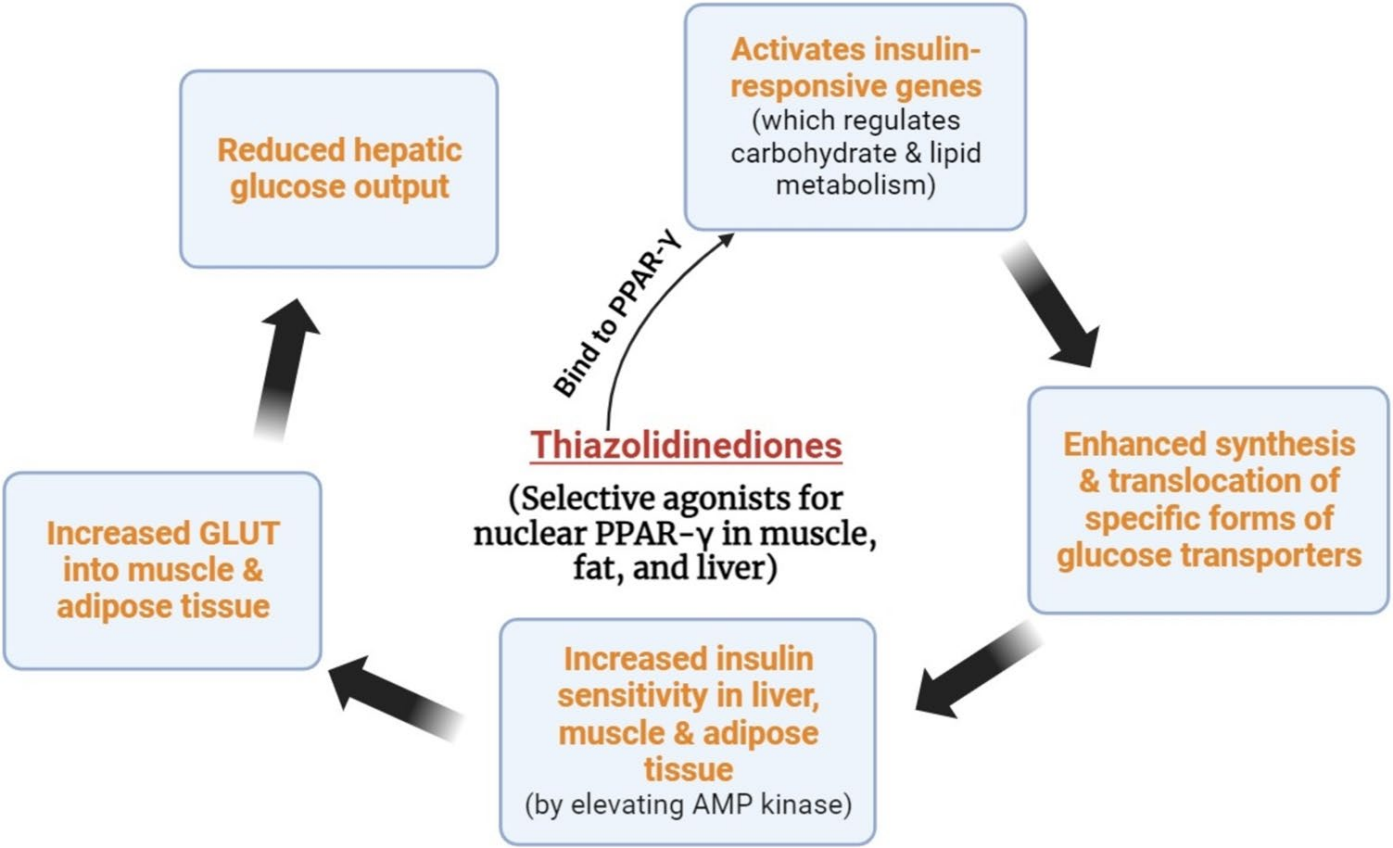
The mechanism of action of thiazolidinediones

As an adjunct to other first-line therapies, thiazolidinediones are increasingly being employed. They stimulate the transcription of genes in response to insulin because they act as agonists for PPAR-γ receptors. The availability of rosiglitazone is limited, with the drug being available in only a few countries, the United States being one of them. Used in the treatment of type 2 patients with insufficient control of hyperglycemia in conjunction with other oral antidiabetic medications and/or insulin, contraindicated in type 1 diabetics (Feingold 2022). The action mechanism of thiazolidinediones is illustrated in Fig. 2.

The main side effects of thiazolidinediones are mild anemia and edema (due to fluid retention), increased risk of HF, weight gain (fluid retention and fat accumulation, also reduced leptin level leading to an increase in appetite and food intake), risk of cardiac failure and myocardial infarction (Singh et al. 2024), risk of hepatotoxicity (it was outlawed then), and risk of urinary bladder cancer (“pioglitazone” has been banned in a few countries) (Singh et al. 2024). However, because of a lack of relevant evidence, the prohibition was repealed in a few months in some of those countries (Pai and Kshirsagar 2016).

#### Imeglimin (AMPK direct activators)

The AMPK is widely expressed in numerous cells and tissues, including the heart, brain, skeletal muscles, and liver. It functions as an intracellular fuel-sensing enzyme, linking energy sensing to metabolic regulation and promoting better energy balance in cells (Ramu et al. 2022).

The main mechanisms of action of imeglimin are that imeglimin increases AMPK in response to activation by upstream kinases, increases glucose absorption and lipid oxidation reaction in the liver and the skeletal muscles while decreasing glycogenesis and lipid synthesis, improves insulin resistance, and has a profound influence on cell energy metabolism (Li et al. 2024); it plays a key role in regulating a variety of other physiological processes, including cell proliferation, biogenesis, and mitochondrial function; affects physiological events linked to IR, including inflammation and endoplasmic reticulum stress, by phosphorylating critical enzymes and transcriptional activators (Garcia and Shaw 2017); and sparks interest as a possible therapeutic target for metabolic disorders, including T2DM because of its critical function in maintaining energy balance.

#### Protein tyrosine phosphatase 1B (PTP 1B) inhibitors

Protein tyrosine phosphatase 1B (PTP 1B) is a major non-transmembrane phosphotyrosine phosphatase found in a variety of tissues and cells. PTP 1B is currently being touted as a viable therapeutic target in the treatment of diabetes. Pancreatic β-cells, leptin signal transduction, and endoplasmic reticulum stress are all involved in PTP 1B’s effect on blood glucose levels.

The mechanisms of action of PTP 1B inhibitors are that they regulate cell development, intercellular signal transduction, gene transcription, and immunological response, among other things (Owen et al. 2013); act on pancreatic β-cells, leading to promoting their proliferation and differentiation while inhibiting apoptosis; and increase insulin sensitivity without compromising euglycemia which works by blocking the negative insulin signaling pathway mediated by PTP 1B(Abdelsalam et al. 2019).

PTP 1B, on the other hand, decreases pancreatic cell proliferation by inhibiting insulin signal transduction (Coronell-Tovar et al. 2024). Moreover, apoptosis in the pancreas is regulated in part by PTP 1B via altering the expression of several signaling pathways. In contrast, leptin signal transduction is altered by PTP 1B, which dephosphorylates and inactivates leptin-activated JAK2(Qian et al. 2016).

#### Sparc-related modular calcium-binding protein-1

Hepatocytes create hepatokines, which regulate metabolism by autocrine, endocrine, and paracrine signaling. SMOC-1 regulates glucose homeostasis. After 4 weeks, once-weekly intraperitoneal injections of recombinant Sparc-related modular calcium-binding protein 1 protein increased insulin sensitivity and glucose tolerance in db/db mice. Human medicinal research is conceivable(Chen et al. 2020). The main mechanism of action of SMOC-1 is that it inhibits hepatic cAMP.

### Insulin secretagogues

#### Drugs acting on sulfonylurea receptors

These drugs work by stimulating pancreatic cells to produce more insulin by binding to the sulfonylurea receptor (SUR) on the ATP-sensitive potassium channel (Lv et al. 2020). Chlorpropamide, tolbutamide, acetohexamide, and tolazamide are first-generation sulfonylureas, while glipizide, glibenclamide, and glimepiride are second-generation sulfonylureas (Sahin et al. 2024). The need for increased potency, a faster start of the action, shorter plasma half-lives, and a longer duration of action led to the development of second-generation sulfonylureas. Sulfonylureas are used only when patients have at least 30% functional beta cells.

As a derivative of benzoic acid, metiglinide serves as the model molecule for the glibenclamide class of non-sulfonylureas. The effects of meglitinides are both shorter-lasting and more immediate. At present, they are used after pregnancy. In contrast, research into newer formulations is underway to determine whether or not they can increase the duration of action and decrease the frequency of administration, so making them appropriate SU substitutes(Chaudhury et al. 2017). Due to the potential for additive effects, the use of meglitinides and sulfonylureas should be avoided(Bereda 2022).

In the same category, repaglinide: *t*1/2 = 1 h, duration of action = 5–8 h, metabolized to inactive derivatives, and its major side effect is hypoglycemia. Nateglinide: t1/2 = 1.5 h, duration of action is less than 4 h, and it is effective whether used alone or in conjunction with non-secretagogue oral medications (like metformin). Glyburide (minimum placental transfer) is a relatively safe substitute for insulin in treating pregnant women with diabetes (Hemmingsen et al. 2016).

The mechanism of action includes activation of insulin production from pancreatic beta cells via ATP-sensitive K^+^ channel blockage, leading to depolarization and Ca^2+^ flow (Tomlinson et al. 2022), lowering hepatic glucose production, and greater peripheral insulin tolerance. However, the side effects include weight gain, hyperinsulinemia, hypoglycemia, and postponed drug elimination and drug buildup in patients with hepatic or renal impairment.

### Glucokinase activators

These drugs work by activating the enzyme glucokinase, which in turn increases glycogen production, glucose metabolism, and insulin secretion. Glucokinase activators can increase pancreatic-cell activity and lower HbA1c levels. Patients with TIIDM who took piragliatin in phase-2b studies saw a dose-dependent reduction in both fasting and postprandial plasma glucose (Seetharaman et al. 2022). Because glucokinase has a low affinity for glucose, it is active at higher glucose levels. This means there is a lesser chance of hypoglycemia. However, investigations on glucokinase activators have found that hypoglycemia still occurs and that the effects last just 4 months, so their long-term usefulness is still questionable (Rosenfeld and Thornton 2023).

### Incretin mimetics (GLP-1 agonists) and DPP-IV inhibitors

The incretins or peptides generated from the gut are glucagon-like peptides (GLPs) and glucose-dependent insulinotropic polypeptides (GIPs). GLP-1 secretion is analogous to insulin secretion from pancreatic cells (Sun et al. 2017). Incretin mimetics lower blood glucose by boosting insulin production and suppressing glucagon release, which helps to lower HbA1c levels (Holst 2019). DPP-IV inhibitors, like incretin mimetic drugs, have a similar effect (Singh et al. 2017).

#### GLP-1 agonists or analogs

The presence of an alanine residue at the N terminal causes GLP-1 to be metabolized by DPP-IV. As a result, additional GLP-1 analogs were created by replacing the alanine group with other amino acids such as threonine, glycine, and serine. GLP-1 analogs were not only stable but also twice as potent as GLP-1 (Wang et al. 2022). The first GLP-1 analog having a glycine residue at the N terminus was “exenatide.”

Exenatide is utilized as an adjuvant to therapy in patients with type 2 diabetes who have failed to maintain optimal glycemic control on metformin, sulfonylurea, glitazone, etc. and demonstrates 53% similarity to human GLP-1 and is resistant to DPP-IV (Seetharaman et al. 2022).

Lixisenatide (Lyxumia®) is taken once daily. Liraglutide (Victoza®) is taken once daily. Dulaglutide (Trulicity®) is taken once weekly (Yu et al. 2018). New evidence suggests that co-infusion of GIP and GLP-1 has a synergistic effect that outperforms solo GLP-1 agonist treatment. Tirzepatide showed noninferiority and superiority to semaglutide for HbA1c reduction at all dose levels studied (Frias et al. 2018).

The main mechanism of action includes that GLP-1 stimulates insulin release by increasing β-cell mass, inhibiting glucagon secretion, slowing gastric emptying, and decreasing appetite. The half-time (*t*_1/2_) of the GLP-1 is equal to 1–2 min, rapidly metabolized by dipeptidyl peptidase IV (DPP-IV).

#### Dipeptidyl peptidase-IV (DPP-IV)

**Fig. 3:**
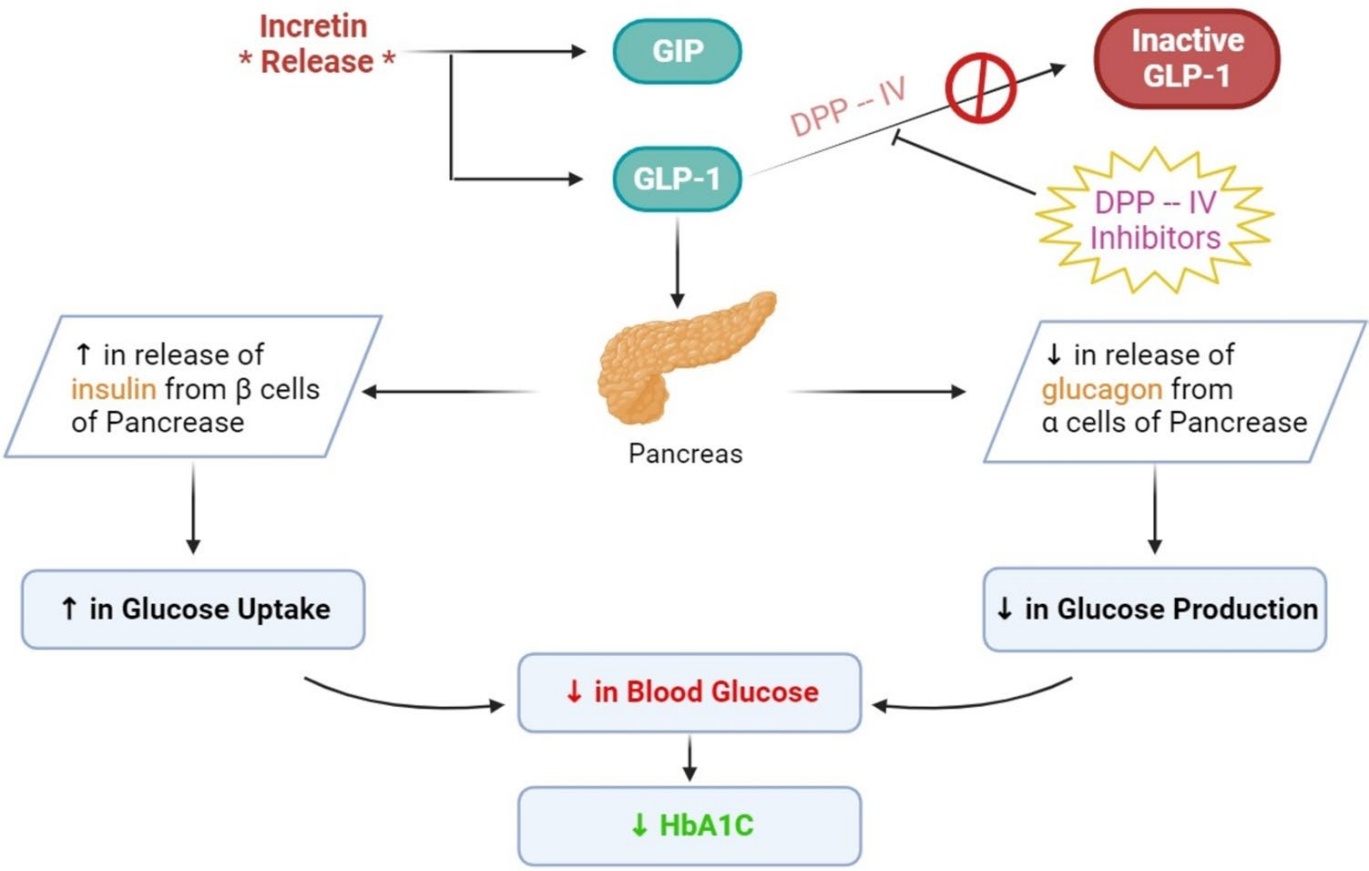
The role of incretin mimetics in decreasing blood glucose and HbA1C

It is a serine protease that exists in two forms: membrane-bound and plasma-soluble (Zare et al. 2024). Due to the inactivation of DPP-IV, GLP-1 has a longer half-life, resulting in greater insulin release in response to meals and a decrease in inappropriate glucagon secretion (Fig. 3). Due to their high oral bioavailability, the following DPP-IV inhibitors are currently available on the market: sitagliptin, vildagliptin, saxagliptin, linagliptin, alogliptin, gemigliptin, anagliptin, teneligliptin, alogliptin, trelagliptin, and omarigliptin. They can be combined with metformin, thiazolidinediones, or sulfonylureas (Gallwitz 2019).

### Free fatty acid receptor 1 (FFAR1) (an emerging target for the treatment of T2DM)

FFAR1 is highly expressed in pancreatic β-cells. GPR40 agonists are currently classified into four groups: thiazolyl derivatives, phenoxy acetamide derivatives, pyrrolyl analogs, and propionic acid derivatives. TAK-875, created by Takeda Pharmaceuticals, was the only agonist that made it to phase 3 clinical trials; however, it was also abandoned due to liver toxicity. As a result, there are several agonists for the receptor’s various binding sites, but no medication is currently available. Drugs that target FFAR1 appear to offer a glimmer of hope; however, additional research into drug development is needed to successfully control type 2 DM (Li et al. 2018).

The mechanism of action includes that intracellular glucose metabolism speeds up as blood glucose levels rise, depolarizing the membrane and shutting the ATP-dependent potassium channel. After that, the voltage-dependent Ca^2+^ channel is opened. Following that, free fatty acid binding to GPR40 enhances glucose-dependent insulin secretion by promoting extracellular Ca^2+^ influx via the phosphatidylinositol signal transduction pathway, which further elevates intracellular Ca^2+^ concentration (Sánchez-Alegría et al. 2021). Moreover, FFAR-1 acts on enteroendocrine cells in the gastrointestinal tract, in addition to directly stimulating insulin production from pancreatic β-cells, which its activation enhances the release of incretins, activates GLP-1 receptors, and boosts insulin secretion indirectly (Mazhar et al. 2023).

The main advantage of FFar1 is that it minimizes the risk of hypoglycemia.

### Glucose regulators

#### Sodium-glucose cotransporter 2 (SGLT2) inhibitor (daglitazone, englenet, ruglietin, caglione, and eglitoglione)

The new class of hypoglycemic medications does not rely on the insulin production pathway. Epithelial glucose transport relies on SGLT1 and SGLT2. SGLT2 handles 97% of kidney tubular glucose reuptake, while SGLT1 handles 3% of gut glucose uptake (Carbó and Rodríguez 2023).

This class’s mechanism of action is that SGLT2 inhibitors bind GLUT and competitively reduce tubular reabsorption of glucose, helping urine excrete excess glucose to restore euglycemia (Nespoux and Vallon 2018). However, the side effects include hypoglycemia, ketoacidosis, and urogenital system infection (Wanner and Marx 2018).

#### 11β-hydroxysteroid dehydrogenase (11β-HSD) inhibitors

11-HSD1, which is widely expressed in metabolic tissues such as the pancreas, liver, and fat, converts cortisone to cortisol and enhances glucocorticoid activity (Mohammed et al. 2021; Wahab et al. 2023). 11-HSD1 inhibitors were well tolerated in clinical trials, and they improved lipid profile, glycemic control, and blood pressure, as well as causing modest weight loss. As a result, medications like VTP-34072, HIS-388, EQ-1280, CNX-010, and others are being developed, and they could be excellent diabetic treatments.

## Chinese traditional medicinal plants

Rai et al. (2019) tested the active ingredient tetramethylpyrazine from *Ligusticum chuanxiong* in a streptozotocin rat model. These drugs’ beneficial effects on glycemic profile, serum insulin, lipid profile, and proinflammatory cytokines point to the potential of a natural molecule targeting the PI3K/Akt/Glut-4 pathway.

Han et al. (Han et al. 2020) investigated the flavonoids and alkaloid contents of *Mulberry* leaf water extract in a diabetic mouse and found that they inhibited α-glucosidase activity. It also lowered free fatty acid (FFA), TNF-α, insulin, and glycosylated serum protein levels in the blood, alleviated kidney impairment, and restored intestinal flora. Mulberry leaf polyphenols (MLPs), a polyphenol derived from mulberry leaves, were found to effectively block glucose transport in a time and dose-dependent manner, according to Li et al. (2020).

*Dendrobium officinale* significantly affected the glucagon-mediated cAMP-PKA and Akt/FoxO1 signaling pathways, promoting hepatic glycogen synthesis while inhibiting hepatic glycogen degradation and gluconeogenesis, according to a study conducted by Liu et al. (2020b), indicating the antidiabetic effect of *Dendrobium officinale* and the associated mechanisms.

### New techniques for DM therapy

#### Stem cell therapy

Stem cell therapy has been shown to have therapeutic potential in the regeneration of insulin-producing cells and immunomodulation of β-cells (Refaie et al. 2021). Over the last two decades, stem cell therapies for T1DM and T2DM have shown encouraging results. All stem cell therapy methodologies focus on mesenchymal stem cell (MSC) therapy, induced pluripotent stem cell (iPSC) therapy, and human pluripotent stem cell (hPSCs) therapy.

#### iPSC therapy

The advantages of this type of treatment technique are that induced pluripotent stem cells (iPSCs) match genetically with most patients, preventing immune reactions from starting, and pluripotency and expansion capacity are infinite (Zang et al. 2017), and the body’s cells can be reprogrammed to restore damaged tissues (Zang et al. 2017).

#### MSC therapy

Mesenchymal stem cell (MSC) therapy restored islet function in animal models while also reducing insulin resistance and glycemic management, indicating a prospective therapeutic benefit. (Zang et al. 2017).

The main advantages of MSCs are that they help to repair injured tissue by promoting neovascularization and increasing angiogenesis (Guillamat-Prats 2021); the immunomodulation, tissue healing, and IPSC generation potential of bone marrow–derived MSCs (BM-MSCs) have been thoroughly demonstrated, and MSCs from adipose tissue, the placenta, and the amnion can be differentiated into functionally competent IPSCs (Zang et al. 2017). One more additional advantage is that umbilical cord-derived MSCs (UC-MSCs) are an interesting alternative source of stem cell therapy due to their ease of procurement, low immunogenicity, increased resemblance to embryonic stem cells (ESCs), and painless procedure.

Although there might be some really interesting advantages, there might be also some challenges to this therapeutic technique which are patients’ tumorigenic risk following transplantation, efficiency of differentiation into β-cells, and patients’ poor survival rate and immunogenicity, and the development of pancreatic-like cells derived from hESC/iPSC in vitro is still a topic of debate.

#### Gene therapy

Gene therapy is the process of inserting exogenous genetic material manipulated in a lab into a target cell using a vector for the therapeutic purpose of disease correction (Grisch-Chan et al. 2019).

##### T1DM management (ectopic insulin production by non-β-cells and the transplanting of genetically engineered islets)

Because of their high capability for ex vivo transduction of human islets, adenoviral vectors are routinely employed (Arutyunyan et al. 2020; Pellegrini et al. 2022). Anti-apoptotic substances such as hepatocyte growth factor (HGF) can help islets survive by inhibiting the immunological response that destroys them, such as interleukin-1 receptor antagonist protein (IRAP) (Jimenez and Bosch 2024). Before transplantation in vivo, adeno-associated vectors (AAV) are employed to convey immunomodulatory genes to the islet graft recipient. It may be observed that autoimmune recurrence and allogeneic rejection can be avoided in diabetic mice (Caldara et al. 2023). Surrogate cells are used to create insulin in gene therapy for ectopic insulin production by non-β-cells; hence, new proteolytic sites in the proinsulin molecule have been developed which will be recognized by the widely spread protease (furin).

##### Types of cells that can be used to achieve ectopic production of insulin

Fibroblasts, myotubes, hepatocytes, and gut K cells are all examples of cell types (Gheibi et al. 2020). In terms of the expression of glucose-sensing molecules such as GLUT-2, hepatocytes are similar to β-cells. Hepatocytes also play a role in carbohydrate metabolism and the regulation of numerous genes expressed in the liver that are regulated transcriptionally by insulin or glucose.

##### T2DM management (amelioration of insulin resistance)

The adenoviral vectors encoding GLP-1 are given systemically to diabetic obese mice. As a result, the mice had increased insulin sensitivity by re-establishing insulin signaling in peripheral tissues and reduced hepatic gluconeogenesis (Saini 2022). In T2DM high-fat diet (HFD) fed rats, systemic AAV-mediated gene transfer of the serine protease kallikrein reversed insulin resistance (Jimenez and Bosch 2024). The peptide hormone bradykinin is a result of kallikrein conversion, finally leading to enhancing insulin sensitivity and consequently accelerating glucose absorption in vivo (Barić and Dobrivojević Radmilović 2021). Furthermore, hexokinase-II (HK-II) gene transfer to white adipose tissue is a promising method for improving insulin sensitivity in T2DM, which can boost glucose uptake in adipocytes (Rabbani and Thornalley 2024).

The advantages of gene therapy include that it is a one-time treatment option; ectopic insulin generation by non-β-cells has progressed due to technological advancements, and it has a better risk–benefit ratio in comparison to lifetime insulin injection and islet transplantation.

This technique includes a lot of challenges to achieve its therapeutic goals which include controlling insulin expression based on a patient’s physiological blood glucose level that requires improvement, urging the development of a cost-effective, long-term therapeutic solution, and more progress is needed for a more tissue-specific response.

### Gene silencing by antisense oligonucleotide and siRNA-based therapy

#### AOs

Antisense oligonucleotides (AOs) are synthetic nucleic acids that regulate gene expression by binding to mRNA targets and stopping their translation into protein (Bhakta and Tsukahara 2020; Barresi et al. 2022).

An example of AOs is IONIS-GCGRRx, which is an antisense oligonucleotide modified with 20-O-methoxyethyl and used to boost GLP-1 levels in a dose-dependent manner by binding selectively to the pre-mRNA of the human glucagon receptor (GCGR) and promoting its destruction by RNase H1. Decreases in HbA1c were observed with IONIS-GCGRRx. Whether as a monotherapy or in addition to other antidiabetics, such as metformin, more research is needed with larger sample sizes (Morgan et al. 2019). The main advantage of AOs is that this therapeutic technique offers target specificity.

#### siRNA therapy

Ribosomal RNA (rRNA), mRNA, and tRNA are the three forms of RNA responsible for protein synthesis, whereas microRNA (miRNA) and small interfering RNA (siRNA) are responsible for gene regulation and expression (siRNA). Because their structure and function in silencing and regulating gene expression are identical, siRNA and miRNA attach to mRNA in a complementary manner. The gene that controls pathogenesis is silenced by siRNA, which prevents the development of diabetes and its complication (Waghode et al. 2024).

##### siRNA in glucose homeostasis

Because glucagon-siRNA (GCGR-siRNA) has a high ability to target the liver, it was encapsulated in lipid nanoparticles and delivered to STZ diabetic mice. GCGR-siRNA (10 mg/kg) considerably reduced blood glucose levels for more than 3 weeks, and oral glucose tolerance was restored (Neumann et al. 2017).

TORC2 which is a coactivator of cAMP-response element-binding protein regulates gluconeogenesis (TORC2). The activity of TORC2 rises in diabetes individuals, which leads to an increase in glucose synthesis via gluconeogenesis. In a rat model, siRNA suppression of the TORC2 gene resulted in successful diabetes treatment. TORC2 was targeted with siRNA supplied in new lipid nanoparticles, resulting in a target-specific effect (Roy et al. 2021).

#### Nanotechnology for insulin delivery

By incorporating nanotechnology and nanoparticles, the hurdles to administering oral insulin (gastric enzymes and limited intestinal permeability) can be overcome.

##### Insulin liposomes

The use of liposomes for insulin administration orally has caught the attention of many researchers. Insulin encapsulated in liposomes is adequately protected against pH changes, enzymatic assault, and immunological detection. Liposomal insulin’s hypoglycemic efficacy is dependent on the lipid component, the charge of its surface, and the physical form of the phospholipid bilayer.

Liposomes with elevated melting dipalmitoyl phosphatidylcholine (DPPC) or negative charge phosphatidylinositol (PI) reduced levels of blood glucose significantly. Kisel et al. created liposomal insulin with both high-melting and negatively charged dipalmitoyl phosphatidylethanol (DPPE) as a lipid component, which demonstrated an elevated plasma insulin and significantly larger hypoglycemic effect in contrast to liposomes composed of natural egg phosphatidylcholine (EPC) (Kisel et al. 2001; Kandregula et al. 2022). Liposomes coated and ligand-mutated have been studied further to increase GI lumen stabilization and boost adherence to the intestinal mucosa for greater insulin hypoglycemic action, respectively (Jash et al. 2021) (Shetty et al. 2023). Furthermore, it was discovered that insulin-loaded biotin liposomal nanoparticles enhanced insulin pharmacokinetic properties as well as hypoglycemic impact, which was due to higher insulin stability and transepithelial absorption through endocytosis(Caturano et al. 2024).

Penetration-enhancing compounds have been added to liposomal insulin to improve insulin penetration across the intestinal mucosa. Niu et al. created “bilosomes” comprising glycocholate that acts as a stabilizer as well as a permeability promoter for oral insulin administration. The lipid-bilayer containing bile salts can shield liposomes against destabilization by biological bile salts inside the GI tract, as well as improve the flexibility of epithelial mucosa for greater vesicle permeation. Liposomes comprising sodium glycocholate (SGC-L) protected insulin from enzymatic degradation by pepsin, trypsin, and a-chymotrypsin much better than those containing its analogs, sodium taurocholate (STC-L) and sodium deoxycholate (SDC-L) (Niu et al. 2011).

##### Insulin nanoemulsion

Nanoemulsions also showed encouraging results in terms of boosting insulin oral bioavailability by shielding insulin from an enzymatic breakdown in the GIT and increasing penetration through the intestinal membrane. Sharma et al. created nanoemulsions with an aqueous phase of insulin, an oil phase of triacetin, a surface active agent of didoceyldimethyl ammonium bromide, and a co-surfactant of propylene glycol. Upon oral delivery, nanoemulsion-loaded insulin demonstrated an improvement in bioavailability when compared to ordinary insulin solution (Santalices et al. 2021). The potential of nanoemulsions coated with alginate/*Aloe vera* gel (A/AV) for the oral administration of insulin in the Caco-2 cell line was investigated. The findings from the cell culture experiments indicate a highly encouraging outcome in terms of insulin translocation across the cell monolayer, with an observed rise of 20 to 25% (Basha et al. 2021).

##### Insulin solid lipid nanoparticles SLNs

Cetyl palmitate-SLNs demonstrated a long hypoglycemic impact and a better relative bioavailability when compared to subcutaneous insulin (Sarangi et al. 2024). Xu et al. created insulin-SLN containing endosomal peptides which showed that oral injection of this insulin-SLN reduced blood glucose levels in diabetics by almost 35% following 3 h and by nearly 20% upon 12 h. In contrast, subcutaneous insulin reduced blood glucose level by 70% after 2 h but restored to its normal level after 12 h (Xu et al. 2018).

CS for coating the SLNs was employed to improve insulin absorption, resulting in a higher relative bioavailability (Mohammadpour et al. 2022).

##### Insulin chitosan nanoparticles

Elsayed et al. created insulin-chitosan nanoparticles containing oleic acid, Plurol oleique as a coformer and Labrasol as a surface active agent, and found that it had a substantial hypoglycemic impact in diabetic rats following oral insulin treatment of 50 IU/kg for 24 h (Elsayed et al. 2009).

In another research, insulin entrapment in chitosan/heparin nanoparticles resulted in typical glucose control. This trial employed a dosage of 60 IU/kg which reduced mean BGL by 30% for 2 h after insulin delivery and persisted for 4 h; it also delivered 50% reduced BGL at 3 h after treatment and persisted for 5 h (Song et al. 2014). Another researcher reported that chitosan insulin nanoparticles contributed to a hypoglycemic activity and a pharmacological bioactivity comparative to subcutaneous insulin (Chellathurai et al. 2023).

##### Insulin alginate nanoparticles

Insulin nanoparticles were synthesized via ionotropic gelation complexation technique of alginate with chitosan followed by cross-linking with calcium chloride, resulting in 748-nm size nanoparticles. The pharmacological action of this preparation in diabetic rats at 50 and 100 IU/kg dosages demonstrated a considerable decrease in blood glucose level by over 40% along with 50 IU/kg and 100 IU/kg doses, with the antidiabetic activity lasting for 18 h (Sarmento et al. 2006).

Insulin may be shielded against deterioration in simulated stomach fluid by alginate/chitosan NPs combined with cationic beta-cyclodextrin polymers (Zhang et al. 2010). In addition, insulin dextran sulfate/chitosan nanoparticles with a diameter of 527 nm were delivered orally at doses of 50 and 100 IU/kg, resulting in a 35% reduction in BGL. The rats were hypoglycemic for more than 18 h (Sarmento et al. 2007).

Ma et al. developed an insulin chitosan-nanoparticle made of chitosan and TPP (tripolyphosphate) with sizes ranging from 269 to 688 nm. When administered orally at insulin levels of 50 and 100 IU/kg, these nanoparticles were efficient in decreasing the glucose level in diabetic rats. The oral insulin dosage of 100 IU/kg, in particular, was capable of keeping the level of glucose stable for 11 h (Ma et al. 2005).

##### Insulin microsphere

Insulin microspheres have been shown in animal studies to maximize the hypoglycemic efficacy of insulin by inhibiting enzymatic degradation of encapsulated insulin and enhancing penetration through epithelial membrane (Sabbagh et al. 2022).

##### Nanoshells and magnetic probes

They are also used to treat DM by injecting nanoshell polymer into the body and warming the skin with a ballpoint pen–sized infrared laser. When the nanoshells are heated, the polymer is activated, causing insulin to be released (Aflori 2021).

##### Inhaler systems

Dry powder insulin can be encapsulated into nanoparticles and inhaled through the lungs for immediate insulin administration to the bloodstream (Liu et al. 2020a).

##### Nano pumps

The pump can maintain blood glucose levels by continuously injecting insulin into the patient’s body. Debiotech has demonstrated the first application of a nano pump with a larger reservoir volume than existing nano pumps (Faro Barros et al. 2022).

##### Nanorobot

Current research is underway to construct a nanorobot with built-in insulin and sensors that can monitor glucose levels on the device’s surface. The sensors can detect an increase in glucose levels and limit the release of insulin when glucose levels are too high (Gandhi and Joshi 2020).

## Therapeutic options for DM with COVID-19

In diabetics, the 2019 new coronavirus illness (COVID-19) can extend both diabetes and COVID-19 consequences. These patients are more likely to develop diabetic ketoacidosis and COVID-19 hyperinflammation. However, the therapeutic techniques of DM might show great effects on these types of patients. Some of the therapeutic effects might include significant anti-coagulant and anti-inflammatory impacts of intravenous insulin (Sardu et al. 2020); the significance of DPP-IV inhibitors in the treatment of SARS-CoV-2 as the virus appears to use DPP-IV as a co-receptor, and pioglitazone mediated the reduction of tumor necrosis factor, interleukin-1, and interleukin-6 secretion, which are associated with hyperinflammation and cytokine release conditions in SARS-CoV-2 patients (Carboni et al. 2020) and lower mortality, associated with taking metformin, which justifies its use in nondiabetic COVID-19 patients in randomized clinical trials (Luo et al. 2020).

## Conclusions and future prospective

Diabetes medication has advanced substantially since the successful isolation of insulin in 1921. Several medications have been proven to improve microvascular and macrovascular outcomes. The development of effective therapeutic options for diabetes that have a better clinical outcome and fewer side effects is the focus of the future. There have been multiple groundbreaking studies on stem cell therapy and gene therapy to date, all of which provide real hope for a cure. In addition, new studies reveal that various therapeutic techniques such as siRNA, nanotechnology, and theranostics hold a lot of promise for improving DM treatment. These new and promising therapeutic techniques could be a huge step forward in diabetes control in the near future. With continuing study and technological advancements, more creative therapeutic techniques will be created, allowing for the effective development of individualized patient care and, ultimately, bringing us closer to a diabetic cure. Several new drug delivery technologies and medicines with unique modes of action are being developed for the treatment of TIDM and TIIDM, with some of them showing sufficient promise in clinical studies or other disorders.

One of the biggest obstacles in gene therapy, which is one of the therapeutic modalities that is expected to entirely cure diabetes, is that fully developed β-cells are not available for insulin replacement therapy. As a result, elucidating the gene regulatory mechanism governing β-cell differentiation will aid in the advancement of gene therapy in clinical practice. Simultaneously, it may aid pancreatic islets in reducing autoimmune responses and improving physiologic function. Functional bioprinting could be used to improve T1DM replacement therapy with these exciting potentials. COVID-19 diabetes presents a therapeutic conundrum in terms of achieving sufficient glycemic control and managing complications. There are numerous hypotheses for managing COVID-19 in diabetics that need to be tested.

## Data Availability

No datasets were generated or analysed during the current study.
